# Nitrogen-water management regimes modulate fatty acid profile, antioxidant defense system, and oxidative status in sorghum (*Sorghum bicolor* L.) silage

**DOI:** 10.1186/s12870-026-09594-4

**Published:** 2026-07-31

**Authors:** Mehmet Alagöz, Mevlüt Türk, Sabri Erbaş, Sercan Önder, Murat Mutlucan, Emre Topçu

**Affiliations:** 1 Department of Field Crops, Faculty of Agriculture, Isparta University of Applied Sciences, Isparta, 32000 Türkiye; 2 Department of Agricultural Biotechnology, Faculty of Agriculture, Isparta University of Applied Sciences, Isparta, 32000 Türkiye; 3 Department of Agricultural Structures and Irrigation, Faculty of Agriculture, Isparta University of Applied Sciences, Isparta, 32000 Türkiye

**Keywords:** Sorghum silage, Nitrogen fertilization, Water stress, Phenolic compounds, antioxidant system, Oxidative stress

## Abstract

Optimizing nitrogen and water management in sustainable silage production is critical for both improving biochemical quality and maintaining plant oxidative balance. This study examined the effects of different nitrogen doses and irrigation levels on the oil content and fatty acid composition, total phenolic content, antioxidant capacity (DPPH and CUPRAC), and antioxidant enzyme activities such as superoxide dismutase (SOD), catalase (CAT) and peroxidase (POD) and oxidative stress activities as malondialdehyde (MDA) and hydrogen peroxide (H_2_O_2_) in sorghum (*Sorghum bicolor* L.) silage. The experiment was conducted in a split-plot design with five nitrogen doses (0, 80, 160, 240, and 320 kg N ha^− 1^) and six irrigation levels (0, 25, 50, 75, 100, and 125% of ET₀). Deficit irrigation applications resulted in increases of 9.42–23.82% in total phenolic content and 1.61–32.26% in antioxidant capacity for DPPH and 4.16–33.33% for CUPRAC compared to full irrigation applications. Medium nitrogen applications (160–240 kg ha^− 1^) strengthened the antioxidant defense system, especially under moderate water stress conditions, showing significant increases in SOD and POD activities. Oil content and unsaturated fatty acid ratio were significantly affected by nitrogen-water interactions. Severe water deficit increased MDA content by 81.81% and H_2_O_2_ accumulation by 32.74%, while the application of appropriate nitrogen doses limited this increase. Multivariate analyses (heat map, PCA, and Pearson correlation) revealed strong positive relationships between total phenolic content and antioxidant capacity, while different correlation interactions were observed between antioxidant enzyme activities and oxidative stress indicators. In conclusion, nitrogen and irrigation management were found to be strategic tools for optimizing biochemical quality and oxidative stability by reshaping the coordination between lipid metabolism and the antioxidant defense system in sorghum silage.

**Supplementary Information:**

The online version contains supplementary material available at 10.1186/s12870-026-09594-4.

## Introduction

Global climate change, rising temperatures and irregular rainfall patterns have made the sustainable use of water resources in agricultural production a strategic priority. Water scarcity, particularly in semi-arid and arid regions, is one of the major stress factors affecting not only the yield of forage crops but also their biochemical quality and nutritional value. Water stress alters carbon partitioning between primary and secondary metabolism by limiting photosynthetic carbon assimilation, leading to significant changes in biochemical composition [1, 2]. These stressinduced changes in carbon allocation constitute a fundamental mechanism underlying plant biochemical adaptation processes.

Sorghum (*Sorghum bicolor* L.) is an important silage crop in semi-arid regions due to its C4 photosynthetic mechanism, high water-use efficiency, and drought tolerance [3, 4]. However, the response of sorghum to stress conditions results in dynamic changes not only at the morphological and physiological levels but also in its biochemical composition. This directly affects the nutritional and biochemical quality of the plant, particularly when used for silage.

Nitrogen indirectly influences phenolic compound and lipid biosynthesis by regulating the carbon-nitrogen balance and directing metabolite allocation between growth and secondary metabolism. While high nitrogen availability promotes growth, moderate nitrogen supply may support phenolic production [5, 6]. Furthermore, low nitrogen availability has been reported to induce the accumulation of phenolic compounds such as flavonols [7] and anthocyanins [8] in various plant species [9–11]. In addition, nitrogen deficiency triggers the formation of reactive oxygen species (ROS), which can damage biomolecules, including membrane lipids [12].

The high phenolic content and antioxidant potential of sorghum have been extensively demonstrated, particularly in its grain and vegetative tissues [13]. Abiotic stress conditions such as drought increase ROS accumulation, whereas antioxidant enzymes including superoxide dismutase (SOD), catalase (CAT), and peroxidase (POD), together with the redox-buffering capacity of phenolic compounds, play important roles in alleviating oxidative stress [14, 15]. The level of oxidative stress is generally assessed using indicators such as malondialdehyde (MDA) and hydrogen peroxide (H_2_O_2_) [16]. However, the combined effects of nitrogen and irrigation regimes on the modulation of the antioxidant defense system remain insufficiently understood.

Oil content and fatty acid composition in forage crops are not only indicators of plant metabolism but also important determinants meat and milk quality. In particular, linoleic (C_18:2_) and *α*-linolenic (C_18:3_) acids are precursors of conjugated linoleic acid formation in milk fat and contribute to improving milk fat quality [17]. Water stress and nitrogen application have been reported to affect carbon metabolism, thereby influencing fatty acid biosynthetic pathways [18, 19].

Although several studies have investigated phenolic content and antioxidant systems in sorghum, research examining the combined effects of nitrogen and irrigation regimes on fatty acid metabolism and oxidative stress responses in silage sorghum remains limited. In particular, little attention has been paid to how nitrogen-water interactions influence the biosynthesis and preservation of polyunsaturated fatty acids, which are critical not only for plant stress adaptation but also for silage nutritional quality and the fatty acid composition of animal-derived products such as milk and meat [20]. Fatty acid composition in silage sorghum is of particular importance because forage-derived polyunsaturated fatty acids directly influence not only plant stress adaptation and membrane stability but also the nutritional quality of animal-derived products. In particular, linoleic (C_18:2_) and α-linolenic (C_18:3_) acids are major precursors of conjugated linoleic acid synthesis in ruminant products and are therefore closely associated with milk and meat quality [21]. Previous studies have demonstrated that agronomic practices such as nitrogen fertilization, harvest management, and environmental stress conditions significantly affect fatty acid biosynthesis and polyunsaturated fatty acid accumulation in forage crops [22, 23]. Moreover, water deficit conditions can alter membrane lipid composition and carbon allocation patterns, thereby modifying fatty acid metabolism in plants. Since silage-based feeding systems directly affect the fatty acid profile of milk and meat, understanding how combined nitrogen–water management regimes regulate fatty acid composition in silage sorghum has considerable importance for both forage quality and livestock nutrition.

Furthermore, previous sorghum studies have generally focused on either physiological growth responses or isolated antioxidant parameters under single stress conditions, whereas the coordinated relationship among fatty acid metabolism, antioxidant defense systems and oxidative stress under combined nitrogen and water management regimes remains insufficiently understood. Therefore, the present study aimed to comprehensively evaluate whether nitrogen supply can modulate oxidative damage and fatty acid biosynthesis under different irrigation regimes by regulating antioxidant defense mechanisms and carbon allocation patterns in sorghum silage.

We hypothesized that nitrogen-water interactions would significantly influence oxidative stress balance, antioxidant enzyme activities, phenolic accumulation, and fatty acid composition, and that optimized nitrogen management under water-limited conditions could improve not only stress tolerance but also the metabolic and functional quality of sorghum silage. Therefore, the present study comprehensively examined the effects of different nitrogen doses and irrigation regimes on oil content and fatty acid composition, total phenolic content, antioxidant capacity, antioxidant enzyme activities and oxidative stress indicators in sorghum silage. In addition, multivariate analyses were employed to elucidate the systematic relationships among biochemical parameters associated with nitrogen-water interactions.

## Materials and methods

### Climate and soil characteristics of experimental site

The study was conducted in Isparta Province (37°45’ N, 30°33’ E; elevation 1035 m), located in the Mediterranean region of Türkiye in 2021. According to the climate data of the experiment area, average temperature and total precipitation during May-September period of 2021 were 20.2 °C and 106.8 mm, respectively, whereas the long-term averages (1929–2020) for the same period were 20.2 °C and 140.0 mm, respectively. The soils of the experimental area had a clay-loam (CL) texture. The soils were slightly saline-alkaline (pH 7.3-8.0), calcareous (approximately 20%), and low in organic matter and nitrogen.

### Irrigation treatments

Irrigation was carried out using a subsurface drip irrigation system. In this system, laterals were installed at a depth of 30 cm with 70 cm spacing below the soil surface using a tractor-mounted burying machine. Six irrigation levels based on field capacity (0% [I_0_], 25% [I_25_], 50% [I_50_], 75% [I_75_], 100% [I_100_], and 125% [I_125_]) and five nitrogen doses (0, 80, 160, 240, and 320 kg N ha^− 1^) were applied. Before initiating irrigation treatments, all plots received the same amount of water through sprinkler irrigation until plants reached a height of 15–20 cm to ensure uniform emergence. The irrigation interval was set at seven days throughout the experiment. The amount of irrigation water applied was calculated using the following equation according to the ratios of the seven-day cumulative reference crop water consumption in the irrigation treatments. Reference crop water consumption values were obtained from an agricultural meteorological station located approximately 500 m from the experimental area and were calculated using the Penman-Monteith method. Irrigation treatments were established based on percentages of accumulative reference evapotranspiration (ET₀) calculated. The irrigation regimes consisted of 0% (I_0_), 25% (I_25_), 50% (I_50_), 75% (I_75_), 100% (I_100_), and 125% (I_125_) ET₀ replacement levels. Therefore, I_25_, I_50_, and I_75_ represented deficit irrigation treatments, I_100_ represented full irrigation, and I_125_ represented excess irrigation (over-irrigation). Seasonal irrigation water amounts applied during the experimental period were 259.7, 346.2, 433.0, 519.6, 606.1, and 692.6 mm for the I_0_, I_25_, I_50_, I_75_, I_100_, and I_125_ treatments, respectively. Irrigation water amounts were measured using water meters installed at the head of each plot (Eq. 1).

1 $$\:\mathrm{I}=\mathrm{A}\:\mathrm{x}\:\mathrm{E}{\mathrm{T}}_{0}\:\mathrm{x}\:\mathrm{P}\:\mathrm{x}\:\mathrm{R}$$

Where, I is the irrigation water amount (Liter), A is the plot area (m^2^), ET_0_ is the cumulative reference crop water consumption amount during the irrigation interval (mm), P is the canopy cover percentage (%) and R is the water restriction ratio (%).

To determine the canopy percentage, plant crown width was measured before each irrigation application and calculated according to Eq. 2 [24].

2 $$\:P=\left(\frac{z}{y}\right)\:\mathrm{x}\:100$$

Where, z represents plant crown width (cm) and y represents row spacing (cm).

### Field experiments

The Greengo sorghum x Sudan grass hybrid cultivar, obtained from Ulusoy Seed Company, was used in the experiment. This annual hybrid is characterized by a high leaf to stem ratio, broad leaves, and a plant height ranging from 250 to 280 cm, making it suited for silage production. In the study, the field trial was conducted using a Randomized Complete Block design (RCBD) with split plots arrangement and three replications. A total of 30 treatments, consisting of five nitrogen doses and six irrigation levels, were evaluated. Nitrogen doses were assigned to the main plots, whereas irrigation treatments were allocated to the subplots. Each plot consisted of five rows spaced 70 cm apart. Plot dimensions were 3.5 m in width, and 8 m in length. A 1.4 m plot space was maintained between the main plots to prevent the movement of water and fertilizer between adjacent plots. In total, 90 plots were established, covering an experimental area of 3494.4 m^2^.

Sowing was performed on May 10, 2021, using a pneumatic precision seeder positioned above the drip laterals. The seeder was adjusted to provide 70 cm row spacing and 8 to 10 cm intra-row plant spacing. Triple superphosphate (TSP, 42% P_2_O_5_) was used as the phosphorus fertilizer. Based on soil analysis results, phosphorus fertiliz was applied at a rate of 80 kg P_2_O_5_ ha^− 1^ at sowing. One third of the nitrogen fertilizer was applied at sowing, while the remaining amount was supplied through two fertigation applications according to the designated treatment doses. Harvesting was carried out on September 20, 2021, when the plants reached the dough stage. To minimize border effects, one outer row from each side of the plot and 1 m from both ends were excluded from the harvest area. The remaining plots were harvested, and yield was determined by weighing the harvested biomass from each plot before sample collection.

### Silage preparation

After harvest, the plant materials were chopped using a single-row tractor-mounted forage harvester, yielding approximately 15 ± 1 kg of green forage per plot. The chopped materials were thoroughly mixed in a container to ensure homogeneity and subsequently ensiled in 25 × 35 cm polyethylene bags with a thickness of 80 μm. Approximately 500 g of each sample was placed into the bags, lightly compressed, vacuum-sealed to remove oxygen, and automatically sealed [25]. To minimize deformation, swelling, or rupture of the bags during the fermentation period, the silage bags were inspected twice weekly during the first month and once weekly thereafter. Vacuum sealing was repeated when necessary for damaged or swollen samples. For each experimental year, a total of 360 silage samples were prepared (6 irrigation treatments x 5 nitrogen doses x 3 replicates x 4 silage replicates). Four silage replicates were prepared for each treatment combination to minimize potential variation associated with the ensiling process and to improve the reliability of silage fermentation and biochemical analyses. Each silage replicate was fermented independently. The silage samples were allowed to ferment at room temperature for 60 days. At the end of the fermentation period, the bags were opened for silage quality measurements and subsequent analyses.

### Sample preparation

Silage samples collected after completion of the fermentation period were dried at 70 °C until constant weight was achieved, ground and prepared for analysis. The prepared samples were analyzed for crude fat content, fatty acid composition, total phenolic content and antioxidant capacity using DPPH and CUPRAC assays.

### Determination of fatty acid components

Crude oil content was determined using a nuclear magnetic resonance (NMR) device (Brüker mq-one, Germany) and expressed as a percentage (%). Oil extraction was performed according to the method described by Howard and Stanley-Samuelson [26]. Briefly, 3 g of dried and ground samples were homogenized in a chloroform-methanol (2:1, v/v), and 50 µL of 2% BHT was added to prevent oxidation. Following centrifugation (4000 rpm, 10 °C, 5 min), the supernatant was collected, and the solvent was evaporated at 40 °C using a rotary evaporator (IKA RV 10 digital). The obtained oil extracts were converted to fatty acid methyl esters (FAME) by incubation in acidified methanol at 85 °C for 90 min and then extracted with hexane. Fatty acid composition was analyzed using a Shimadzu GC-2010 Plus gas chromatography system equipped with a flame ionization detector (FID). Separation was performed using a CP-WAX 52 CB column (60 m × 0.32 mm × 1.20 μm). Helium was used as the carrier gas at a flow rate of 3 mL min^− 1^. Samples (1 µL) were injected at a split ratio of 10:1. The injection and detector temperatures were set at 250 °C and 265 °C, respectively. The initial column temperature was maintained at 80 °C for 4 min, increased to 250 °C at a rate of 3 °C min^− 1^, and then held at 250 °C for 20 min. The total analysis time was 85 min. Fatty acids were identified based on the relative retention times of a commercial FAME standard mixture (Sigma, Supelco^®^ 37-Component FAME Mixture). Fatty acid composition was expressed as the relative percentage of the total fatty acids [27].

### Total phenolic content and antioxidant capacity

Ten grams of dried and ground silage samples were extracted with 100 mL of methanol in a 1 shaker at 100 rpm for 30 min. The mixtures were then kept in a dark at room temperature for 24 h and subsequently filtered. The obtained supernatants were used for the determination of phenolic compounds and antioxidant capacity [28].

Total phenolic content was determined using the modified Folin-Ciocalteu method [29]. Briefly, 0.1 mL of methanol extract was mixed with 2.5 mL of deionized water and 0.1 mL of Folin-Ciocalteu reagent (2 N). After incubation in the dark for 6 min, 0.5 mL of 20% carbonate solution was added. The reaction mixture was further incubated in the dark for 30 min to allow color development. Absorbance was measured at 760 nm using a spectrophotometer (Shimadzu UV-1280, Kyoto, Japan). Results were expressed as mg gallic acid equivalents (GAE) g^− 1^ dry matter (DM).

Antioxidant capacity was measured using CUPRAC and DPPH assays. The DPPH (2,2-diphenyl-1-picrylhydrazyl) assays was performed based on the radical scavenging ability of antioxidants [30]. Briefly, 1 mL of methanol extract was mixed with 1 mL of 99% ethanol and 2 mL of 0.2 mM DPPH solution. After incubation at room temperature in the dark for 30 min, absorbance was measured at 515 nm using a spectrophotometer (Shimadzu UV-1280, Kyoto, Japan). DPPH radical scavenging activity was expressed as mmol Trolox equivalent g^− 1^ DM.

The CUPRAC assay was performed based on the reduction of the copper(II)-neocuproine complex [31]. Briefly, 1 mL of Cu(II) chloride (10 mM), 1 mL of neocuprin (7.5 mM), 1 mL of ammonium acetate buffer (1 M), and 0.6 mL of deionized water were added to 0.5 mL of methanol extract. After incubation at room temperature for 30 min, absorbance was measured at 450 nm against a reagent blank using a spectrophotometer (Shimadzu UV-1280, Kyoto, Japan). The results were expressed as mmol Trolox equivalents g^− 1^ DM.

### Antioxidant enzyme activity

For the determination of SOD, POD, and CAT activities, 2 g of fresh silage samples were ground in liquid nitrogen and homogenized in 100 mM cold sodium phosphate buffer (pH 6.4) containing 0.5% polyvinylpyrrolidone (PVPP). The homogenates were centrifuged at 15,000 rpm for 20 min at 4 °C, and the resulting supernatants were used for enzyme activity assays.

SOD activity was determined based on the inhibition of nitroblue tetrazolium (NBT) reduction [32]. The reaction mixture consisted of 2.8 mL reaction solution containing 0.1 mM EDTA, 13 mM methionine, 50 mM sodium carbonate, 75 µM nitroblue-tetrazolium and 50 mM sodium phosphate buffer (pH 7.8), together with 0.1 mL of 2 mM riboflavin, and 0.1 mL of enzyme extract. The reaction mixtures were incubated under light for 15 min, and the absorbance was measured at 560 nm a spectrophotometer (Shimadzu UV-1280, Kyoto, Japan). One unit of SOD activity was defined as the amount of enzyme required to inhibit NBT reduction by 50%.

POD activity was determined using guaiacol as the substrate [29]. The reaction mixture contained 0.1 mL of enzyme extract and 2.9 mL of 100 mM phosphate buffer (pH 6.4) containing 20 mM guaiacol and 40 mM H₂O₂. The increase in absorbance (Ɛ_470_ = 26.6 M^− 1^ cm^− 1^) was recorded at 470 nm for 2 min using a spectrophotometer (Shimadzu UV-1280, Kyoto, Japan). One unit of POD activity was defined as the amount of enzyme responsible for the formation of 1 µmol tetraguaiacol min^− 1^ through guaiacol oxidation in the presence of hydrogen peroxide.

CAT activity was determined based on the decomposition rate of H₂O₂ [33]. The reaction mixture consisted of 0.1 mL of enzyme extract and 2.9 mL of sodium phosphate buffer (50 mM, pH 7.0) containing 40 mM H_2_O_2_. The decrease in absorbance (Ɛ_240_ = 36 M^− 1^ cm^− 1^) was recorded at 240 nm for 2 min using a spectrophotometer (Shimadzu UV-1280, Kyoto, Japan). One unit of CAT activity was determined as the amount of enzyme responsible for H_2_O_2_ decomposition min^− 1^.

SOD, POD, and CAT activities were calculated based on protein concentration, which was determined according to the Bradford method [34]. All antioxidant enzyme activities were expressed as U mg protein^− 1^ fresh weight (FW).

### Determination of malondialdehyde and H_2_O_2_

Lipid peroxidation level was determined by measuring malondialdehyde (MDA) content according to the 2-thiobarbituric acid (TBA) [35]. Briefly, 1 mL of sample extract was added to 4.5 mL of a solution containing 0.5% TBA in 20% trichloroacetic acid (TCA). The mixture was heated in a boiling water bath for 30 min and then rapidly cooled in an ice bath. Following centrifuged at 10,000 rpm for 10 min, absorbance was measured at 532 nm and 600 nm using a spectrophotometer (Shimadzu UV-1280, Kyoto, Japan). An extinction coefficient of 155 mM cm^− 1^ was used for MDA calculation.

H₂O₂ content was determined using the potassium iodide method [36]. The reaction mixture consisted of 0.5 mL of 10 mM potassium phosphate buffer (pH 7.0), 1 mL of 1 M potassium iodide, and 0.5 mL of sample extract. After incubation in the dark for 15 min, absorbance was measured at 390 nm using a spectrophotometer (Shimadzu UV-1280, Kyoto, Japan). The H_2_O_2_ content was expressed as µmol g^− 1^ FW.

### Statistical analyses

Experimental data were analyzed using the SAS software package (SAS Institute, 1998) according to a Randomized Complete Block design (RCBD) with split plots. All analyses were performed with three replications. Duncan’s multiple range test was performed at a 5% significance level to compare treatment means. Pearson correlation analysis, principal component analysis (PCA), and heatmap analysis were conducted using the RStudio v4.3.1 [37]. The “devtools”, “RColorBrewer”, and “heatmap” packages were used for the heatmap analysis; the “meta” package was employed for the correlation analysis; and the “FactoMineR”, “factoextra”, and “pca3d” packages were used for principal component analysis.

## Results

**Fig. 1:**
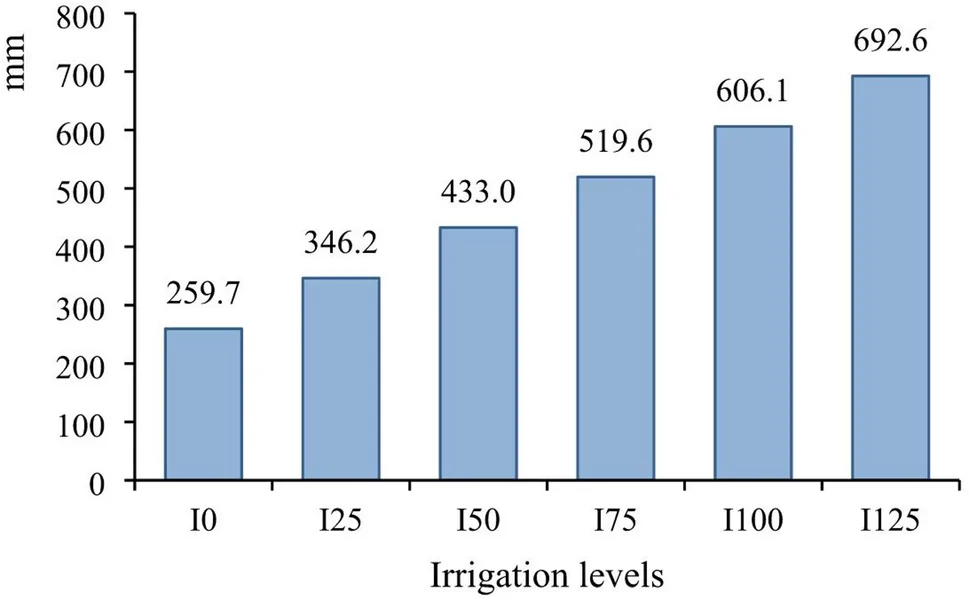
Irrigation water quantities measured during the trial period (mm)

Irrigation water amounts applied during the experimental period varied proportionally according to irrigation treatments and are presented in Fig. 1. The total applied irrigation water ranged from 259.7 mm in the I_0_ treatment to 692.6 mm in the I_125_ treatment, indicating a clear gradient from severe water restriction to over-irrigation among the treatments.

### Crude oil content

**Fig. 2:**
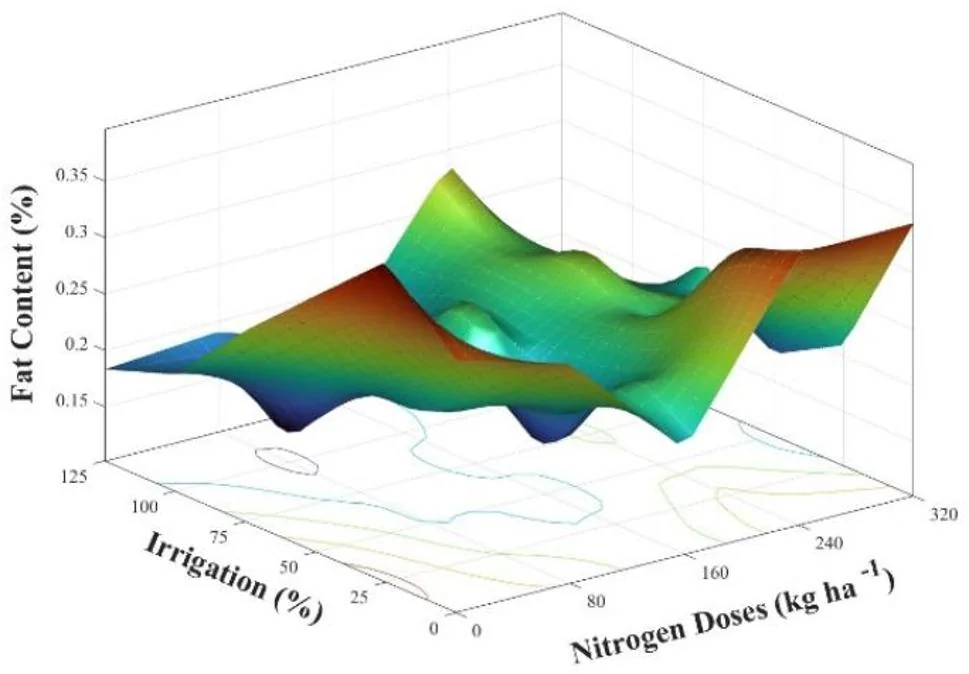
3D response surface showing the combined effects of nitrogen dose (kg ha⁻¹) and irrigation level (%) on fat content of sorghum silage

Crude oil content of sorghum silage was significantly affected by nitrogen dose, irrigation level, and their interaction (Table 1). Average crude oil content ranged from 0.178% to 0.292%. In general, higher oil contents were observed under deficit irrigation conditions (I_0_-I_50_), whereas increasing irrigation levels resulted in a significant reduction in oil content. The lowest crude oil contents were obtained under the I_100_ and I_125_ treatments. Among nitrogen applications, N_0_ and N_240_ treatments resulted in relatively higher crude oil contents, whereas lower values were obtained under N_80_ and N_160_ treatments. The three-dimensional surface graph further demonstrated that crude oil content reached its maximum under deficit irrigation conditions, particularly at low to moderate nitrogen doses (Fig. 2). In contrast, oil content remained low under high irrigation levels regardless of nitrogen doses.

**Table 1:** Effects of nitrogen doses and irrigation levels on fat content, phenolic compounds, antioxidant capacity, antioxidant enzyme activities, and oxidative stress indicators in sorghum silage

Treatments	Fat Content (%)	Total Phenolic Content (mg GAE g^− 1^ DM)	Antioxidant Capacity		Antioxidant Enzymes			Oxidative Stress	
			DPPH (mMol TE)	CUPRAC (mMol TE)	POD (U mg protein^− 1^)	CAT (U mg protein^− 1^)	SOD (U mg protein^− 1^)	MDA (nmol g^− 1^)	H_2_O_2_ (µmol g^− 1^)
Nitrogen Doses									
N_0_	0.265 ± 0.076 a	34.22 ± 4.48 b	0.0055 ± 0.0006 b	0.061 ± 0.007 b	2.05 ± 1.43 d	11.89 ± 2.43 b	36.22 ± 9.47 c	0.426 ± 0.288 a	60.13 ± 11.70 b
N_80_	0.210 ± 0.051 b	33.15 ± 6.12 b	0.0048 ± 0.0009 c	0.058 ± 0.010 c	4.02 ± 1.42 bc	9.03 ± 1.98 d	37.10 ± 13.91 c	0.385 ± 0.234 b	62.63 ± 10.78 a
N_160_	0.221 ± 0.062 b	33.60 ± 3.94 b	0.0046 ± 0.0010 d	0.059 ± 0.008 c	4.26 ± 1.38 b	9.61 ± 2.65 c	45.96 ± 6.84 b	0.142 ± 0.099 c	55.20 ± 6.53 c
N_240_	0.265 ± 0.058 a	38.92 ± 4.31 a	0.0058 ± 0.0011 a	0.067 ± 0.013 a	3.81 ± 1.32 c	7.10 ± 2.86 e	48.74 ± 7.95 b	0.176 ± 0.073 c	55.68 ± 9.95 c
N_320_	0.231 ± 0.070 ab	34.38 ± 4.00 b	0.0058 ± 0.0006 a	0.066 ± 0.010 a	5.44 ± 1.39 a	15.28 ± 2.84 a	56.92 ± 11.48 a	0.080 ± 0.046 d	56.97 ± 7.82 c
Irrigation Levels									
I_0_	0.292 ± 0.060 a	40.68 ± 4.47 a	0.0062 ± 0.0002 a	0.072 ± 0.009 a	5.06 ± 1.25 a	14.07 ± 3.18 a	43.61 ± 5.23 c	0.481 ± 0.288 a	70.06 ± 6.65 a
I_25_	0.282 ± 0.052 a	36.85 ± 4.11 b	0.0061 ± 0.0007 b	0.069 ± 0.006 b	4.92 ± 1.57 a	11.47 ± 3.75 b	47.58 ± 11.37 b	0.348 ± 0.287 b	67.52 ± 7.62 b
I_50_	0.268 ± 0.069 a	36.06 ± 2.83 b	0.0054 ± 0.0010 c	0.067 ± 0.006 b	4.03 ± 1.55 b	10.25 ± 2.79 c	48.65 ± 12.84 ab	0.202 ± 0.111 c	59.97 ± 2.37 c
I_75_	0.223 ± 0.036 b	32.36 ± 3.69 c	0.0050 ± 0.0008 d	0.063 ± 0.008 c	3.47 ± 1.72 c	10.35 ± 2.50 c	51.41 ± 11.00 a	0.174 ± 0.094 cd	54.03 ± 2.04 d
I_100_	0.187 ± 0.039 c	32.19 ± 2.63 c	0.0047 ± 0.0006 e	0.056 ± 0.008 d	3.82 ± 1.66 b	8.33 ± 2.54 e	46.06 ± 10.91 bc	0.158 ± 0.091 d	50.03 ± 3.04 e
I_125_	0.178 ± 0.034 c	30.99 ± 4.54 c	0.0042 ± 0.0005 f	0.048 ± 0.004 e	2.19 ± 1.12 d	9.02 ± 4.88 d	32.62 ± 14.97 d	0.087 ± 0.051 e	47.12 ± 3.90 f
Significance									
Nitrogen Levels	4.91 *	16.64 **	187.55 **	49.90 **	228.44 **	376.25 **	33.81 **	219.21 **	35.62 **
Irrigation Levels	21.14 **	31.68 **	192.74 **	83.53 **	126.79 **	154.09 **	43.11 **	192.55 **	250.21 **
Nitrogen x Irrigation	1.59^ns^	2.69 **	13.44 **	6.81 **	27.97 **	28.29 **	15.09**	28.75 **	8.39 **

Values are means ± SD. Different lowercase letters within the same column indicate significant differences according to Duncan’s multiple range test at *p* < 0.05. *, **, and ns indicate significance at *p* < 0.05, *p* < 0.01, and non-significant, respectively

### Fatty acid composition

GC-FID analyses identified numerous short-, medium-, and long-chain fatty acids in sorghum silage (Table 2). Palmitic acid (C_16:0_) was the main fatty acid in all treatments, followed by linoleic acid (C_18:2_), oleic acid (C_18:1_), and *γ*-linolenic acid (C_18:3_).

**Table 2:** Fatty acid composition (%) and saturation profile (%) of sorghum silage as affected by nitrogen doses and irrigation regimes

Retention Time (min)	Nitrogen Doses	*N*_0_						*N*_8_					
	Irrigation Levels	I_0_	I_25_	I_50_	I_75_	I_100_	I_125_	I_0_	I_25_	I_50_	I_75_	I_100_	I_125_
13.037	Butyric acid (C4:0)	2.56	2.57	6.12	1.23	3.20	1.84	2.10	5.06	1.49	2.22	3.07	2.32
15.969	Caproic acid (C6:0)	0.74	0.82	0.16	1.18	1.21	0.61	1.75	1.25	0.50	1.20	0.86	0.45
18.853	Caprylic acid (C8:0)	1.53	0.31	1.01	1.14	1.18	5.04	0.08	0.32	0.86	1.08	2.10	0.83
24.021	Capric acid (C10:0)	0.46	0.48	0.29	0.62	0.47	0.35	0.46	0.50	0.23	0.40	0.34	0.49
27.523	Undecanoic acid (C11:0)			0.15				0.32					
30.169	Lauric acid (C12:0)	0.68	0.86	0.54	0.85	2.41	0.37	0.51	1.63	0.66	0.84	0.70	0.41
31.939	Tridecanoic acid (C13:0)		0.66	0.43	0.51	0.95	0.30	0.24	0.80	0.15	0.21		
35.986	Myristic acid (C14:0)	0.74	0.84	0.53	1.32	1.61	0.35	0.64	2.02	0.63	0.85	0.68	0.39
38.343	Myristoleic acid (C14:1)		0.49		0.29	0.45			0.39	0.11			0.58
39.720	Pentadecanoic acid (C15:0)		0.34	0.26	0.39	0.25	0.32	0.30		0.34	0.32	0.49	0.22
40.782	cis-10-Pentadecenoic acid (C15:1)	0.65	0.79	1.14	1.08	0.98	2.31	0.87	0.83	0.88	1.01	1.08	1.37
41.492	Palmitic acid (C16:0)	29.23	27.94	26.82	25.82	24.95	22.44	28.60	26.74	25.83	30.16	28.11	28.13
43.435	Palmitoleic acid (C16:1)	1.13	0.41	0.25	0.25	0.10	0.82	0.72	0.50	0.19	0.42	0.82	1.96
45.600	Heptadecanoic acid (C17:0)	0.57	1.27	0.97	1.54	1.11	0.87	0.92	1.48	0.71	0.58	4.16	0.82
46.445	Stearic acid (C18:0)	4.66	4.51	2.76	6.85	3.49	2.67	5.64	4.61	3.08	5.48	1.03	2.16
47.833	Elaidic acid (C18:1)	0.50	1.14	0.37	0.41	0.29	2.01	2.72	1.51	0.37	0.41	0.39	
47.999	Oleic acid (C18:1)	18.59	15.07	15.55	10.89	18.76	15.37	6.70	5.86	15.87	12.13	14.88	16.95
49.051	Linoelaidic acid (C18:2)								0.14				
49.958	Linoleic acid (C18:2)	22.72	22.10	19.01	17.09	14.30	12.62	13.95	14.79	23.62	20.92	19.59	19.91
51.035	Arachidic acid (C20:0	0.53	1.14	0.38	0.61	0.58	0.93	2.95	2.21	0.88	0.97	0.85	1.14
52.025	γ-linolenic acid (C18:3)	7.11	7.35	15.49	14.26	10.36	18.22	10.07	14.86	11.97	11.26	14.61	14.16
53.939	Linolenic acid (C18:3)					0.54	0.53				0.27		
55.096	Heneicosanoic acid (C21:0)					0.84				0.86	0.27		0.26
55.788	Eicosadienoic acid (C20:2)	1.08	1.20		0.86	0.69	0.72	3.25	2.62	0.63	0.85	1.00	0.26
57.101	Eicosanoic acid (C20:0)					0.10		0.50					
57.924	Arachidonic acid (C20:4)	1.68			3.58	0.90			0.18	1.59	1.58	1.37	
58.608	Tricosanoic acid (C23:0)		1.28				2.36	2.87	1.49				
60.068	cis-13.16-Docosadienoic acid (C22:2)									0.37			
60.333	Lignoceric acid (C24:0)	1.00	1.73	0.84	1.07	1.25	0.76	2.88	2.47	1.65	1.11	1.04	0.60
61.805	Nervonic acid (C24:1)			0.58	0.74	0.18	0.47		0.11	0.66	0.66		
65.310	Docosahexaenoic acid (C22:6)	0.76	1.02				0.24	0.35	0.14	0.19	0.22	0.61	0.13
Other fatty acids		3.08	5.68	6.35	7.42	8.85	7.48	10.61	7.49	5.68	4.58	2.22	6.46
Saturated Fatty Acids (SFA)		42.70	44.75	41.26	43.13	43.60	39.21	50.76	50.58	37.87	45.69	43.43	38.22
Monounsaturated Fatty Acids (MUFA)		20.87	17.90	17.89	13.66	20.76	20.98	11.01	9.20	18.08	14.63	17.17	20.86
Polyunsaturated Fatty Acids (PUFA)		33.35	31.67	34.50	35.79	26.79	32.33	27.62	32.73	38.37	35.10	37.18	34.46

Retention Time (min)	Nitrogen Doses	*N*_16_							*N*_24_							*N*_32_						
	Irrigation Levels	I_0_		I_25_	I_50_	I_75_	I_100_	I_125_	I_0_		I_25_	I_50_	I_75_	I_100_	I_125_	I_0_		I_25_	I_50_	I_75_	I_100_	I_125_
13.037	Butyric acid (C4:0)	5.15		13.09	1.40	4.35	6.56	4.86	4.16		1.25	3.66	4.42	3.29	2.89	4.10		4.65	4.88	2.42	6.86	2.45
15.969	Caproic acid (C6:0)	3.05		1.19	0.80	2.49	0.62	1.54	0.61		0.51	0.87	1.49	0.91	0.58	1.68		0.87	0.94	1.75	1.27	0.81
18.853	Caprylic acid (C8:0)	2.43		1.10	0.57	1.81	0.84	1.62	0.75		0.75	1.78	1.61	1.33	1.64	0.88		1.30	0.84	1.75	0.96	1.37
24.021	Capric acid (C10:0)			0.58	0.21		0.47				0.38	0.48	2.60	1.63	0.19			1.55	0.28	2.31		0.30
27.523	Undecanoicacid (C11:0)			0.17	0.14		0.27		0.31				1.38			0.59				0.50		
30.169	Lauric acid (C12:0)	1.21		0.86	0.65	1.55	0.51	1.48	0.65		0.64	0.72	1.23		0.37	1.69		0.60	0.63	0.35	0.37	0.62
31.939	Tridecanoic acid (C13:0)			0.44	0.42	0.92	0.21	1.01			0.39	0.24	2.46	0.87						0.78		0.23
35.986	Myristic acid (C14:0)	0.66		0.95	0.56	0.71	0.43	0.35	0.73		0.75	0.58	1.23	0.52	0.38	0.54		0.54	0.64	0.67	0.36	0.57
38.343	Myristoleic acid (C14:1)			0.37		0.49	0.34	0.58					0.55	1.88	0.24					0.51		
39.720	Pentadecanoic acid (C15:0)	1.28		0.34	0.19		0.26	0.23	0.26		0.34	0.84	1.06	1.15	0.52			0.27	0.28	0.44	0.52	0.90
40.782	cis-10-Pentadecenoic acid (C15:1)			0.80	0.82	1.58	1.37	1.84	0.62		1.26	2.02		1.57	1.12	1.72		0.79	1.19	1.57		2.13
41.492	Palmitic acid (C16:0)	30.18		26.01	21.49	25.05	25.39	25.76	23.65		25.61	26.40	28.30	28.15	27.88	23.92		24.59	24.57	23.33	18.71	25.55
43.435	Palmitoleic acid (C16:1)				0.10		0.56	0.81	0.70		1.11	0.97		0.25	0.67	0.67			0.18	0.26		1.33
45.600	Heptadecanoic acid (C17:0)	0.64		1.63	0.82	1.23	0.76	1.06	0.40		1.45	0.86		0.24		1.80		1.65	0.46	1.10	1.76	0.56
46.445	Stearic acid (C18:0)	2.60		5.14	2.84	2.75	1.35	2.47	3.39		3.05	2.94	1.43	2.61	2.99	3.51		2.84	2.83	3.33		3.17
47.833	Elaidic acid (C18:1)	0.40			0.35	0.97	0.31	3.69	0.40		0.39	0.46	2.37	0.22	2.55	3.12		1.98	0.33			
47.999	Oleic acid (C18:1)	10.21		10.20	18.85	10.37	15.29	10.01	16.62		11.45	10.87	9.26	12.73	14.11	9.37		12.15	16.97	7.55	24.41	9.45
49.051	Linoelaidic acid (C18:2)					0.83										0.88						
49.958	Linoleic acid (C18:2)	15.90		18.27	24.93	18.20	18.13	15.04	23.97		21.25	18.94	14.36	18.81	20.99	15.62		16.21	22.54	18.59	29.75	17.86
51.035	Arachidic acid (C20:0	0.87		0.72	0.58	0.54	0.49	0.66	0.62		1.00	0.63		0.23		1.42				3.54		0.55
52.025	γ-linolenic acid (C18:3)	15.19		12.43	10.66	10.25	16.84	15.83	10.82		14.23	17.95	11.79	15.84	16.55	13.89		15.44	13.27	16.26	11.05	23.17
53.939	Linolenic acid (C18:3)	1.72			0.61	0.55	0.50		0.82		0.74				0.38	1.28		0.35	0.19			
55.096	Heneicosanoic acid (C21:0)			0.21	0.75		0.38		0.27		0.24	0.41										
55.788	Eicosadienoic acid (C20:2)			0.72	0.65	2.55	0.30	0.83	0.43		0.45	0.38	0.64	0.55	0.58			0.20	1.62	1.52		
57.101	Eicosanoic acid (C20:0)								0.28				0.42		0.44							
57.924	Arachidonic acid (C20:4)			2.15	2.45		1.34	0.24			1.32	2.31						2.49	0.98	1.05		
58.608	Tricosanoic acid (C23:0)					4.90	1.44	3.39	2.75				1.77	1.87		2.96						2.10
60.068	cis-13.16-Docosadienoic acid (C22:2)																					
60.333	Lignoceric acid (C24:0)	2.10		0.08	1.13	2.22	0.81	1.14	0.69		1.38		0.84	0.76	0.45	0.90		1.15	1.37	2.87	0.54	1.14
61.805	Nervonic acid (C24:1)			0.30	0.23				0.71		0.26			0.54	0.29				0.71			
65.310	Docosahexaenoic acid (C22:6)			0.23	0.12		0.19	0.26			0.23		1.72								0.94	
Other fatty acids		6.41		2.02	7.68	5.69	5.04	5.30	5.39		9.57	5.69	9.07	4.05	4.19	9.46		10.38	4.30	7.55	2.50	5.74
Saturated Fatty Acids (SFA)		50.17		52.51	32.55	48.52	40.79	45.57	39.52		37.74	40.41	50.24	43.56	38.33	43.99		40.01	37.72	45.14	31.35	40.32
Monounsaturated Fatty Acids (MUFA)		10.61		11.67	20.35	13.41	17.87	16.93	19.05		14.47	14.32	12.18	17.19	18.98	14.88		14.92	19.38	9.89	24.41	12.91
Polyunsaturated Fatty Acids (PUFA)		32.81		33.80	39.42	32.38	37.30	32.20	36.04		38.22	39.58	28.51	35.20	38.50	31.67		34.69	38.60	37.42	41.74	41.03

Palmitic acid content ranged from 18.71% to 30.18% depending on nitrogen dose and irrigation regime. Values varied between 22.44% and 29.23% in N_0_, 25.83% to 30.16% in N_80_, 21.49% to 30.18% in N_160_, 23.65% to 28.30% in N_240_, and 18.71 to 25.55% in N_320_ treatments. In general, palmitic acid ration decreased with increasing irrigation level. Higher values were predominantly observed under deficit and moderate irrigation conditions (I_0_-I_50_), whereas relatively lower values were obtained under I_100_ and I_125_ treatments. Oleic acid content exhibited substantial variation among treatments, ranging from 5.86% to 24.41%. Values ranged from 10.89% to 18.76% in N_0_, 5.86% to 16.95% in N_80_, 10.01% to 18.85% in N_160_, 9.26% to 16.62% in N_240_, and 7.55% to 24.41% in N_320_ treatments. In most nitrogen applications, higher oleic acid ratios were observed under deficit irrigation, whereas increased irrigation generally resulted in lower values. Linoleic acid was one of the major unsaturated fatty acids identified in sorgum silage and ranged from 12.62% to 29.75%. The range of linoleic acid varied between 12.62% and 22.72% in N_0_, 13.95% to 23.62% in N_80_, 15.04% to 24.93% in N_160_, 14.36% to 23.97% in N_240_, and 15.62% to 29.75% in N_320_ treatments. Higher linoleic acid ratios were generally observed under deficit and moderate irrigation levels, whereas excessive irrigation resulted in a decreasing trend. In addition, medium and high nitrogen doses (N_240_-N_320_) promoted linoleic acid accumulation under specific irrigation regimes. The γ-linolenic acid content ranged from 7.11% to 23.17% and showed marked variation depending on irrigation regime. Values ranged between 7.11% and 18.22% in N_0_, 10.07% to 14.61% in N_80_, 10.25% to 16.84% in N_160_, 10.82% to 17.95% in N_240_, and 11.05% to 23.17% in N_320_ treatments. In contrast to palmitic and oleic acids, γ-linolenic acid rate generally increased with increased irrigation level. Short-chain fatty acids, including butyric, caproic, caprylic, and capric acids, were found at relatively low proportions across all treatments, however, their contents varied considerably depending on nitrogen dose and irrigation regime. Butyric acid content ranged from 1.23% to 13.09%. In some combinations of high nitrogen supply and deficit to moderate irrigation, marked increases in butyric acid were observed, although these increases did not follow a consistent pattern across all treatments.

Overall, saturated fatty acids (SFA) constituted the largest proportion of the total fatty acid profile, ranging from 31.35% to 52.51%. Monounsaturated fatty acids (MUFA) were generally higher under low nitrogen doses (N_0_ and N_80_) and high irrigation regimes. In contrast, polyunsaturated fatty acid (PUFA) were relatively higher at N_0_, N_80_, N_160_, and N_240_ treatments combined with the I_50_ irrigation regime, whereas the highest PUFA rate at N_320_ were observed in the I_100_ and I_125_ treatments. These results indicate that nitrogen and irrigation regimes substantially influenced the saturation profile of fatty acids in sorghum silage.

### Total phenolic content

**Fig. 3:**
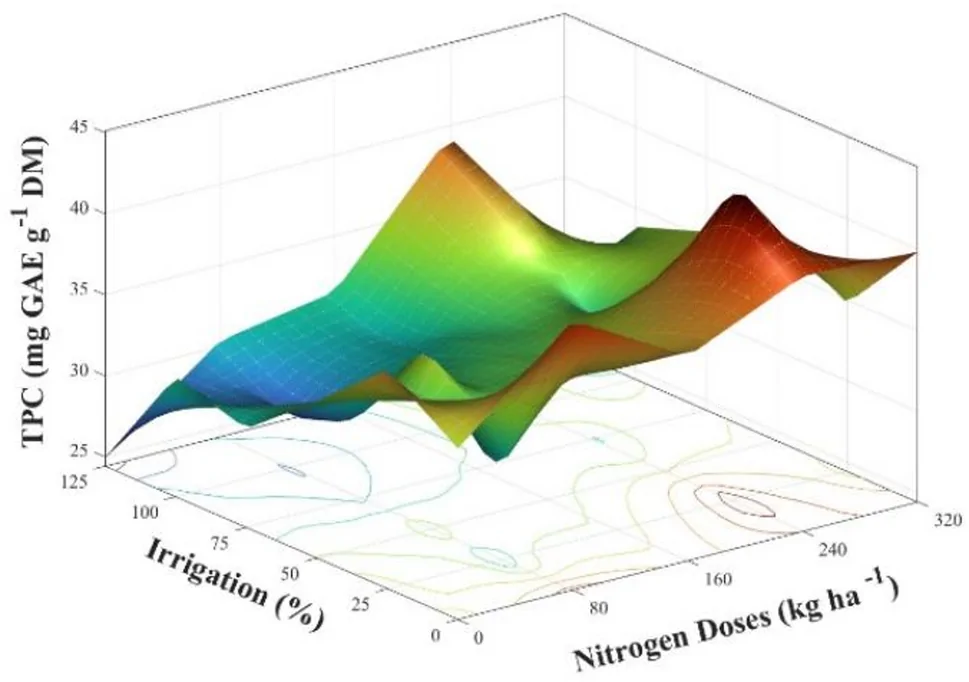
3D response surface showing the combined effects of nitrogen dose (kg ha⁻¹) and irrigation level (%) on total phenolic content in sorghum silage

Total phenolic content was significantly affected by nitrogen dose, irrigation level, and their interaction (*p* < 0.01; Table 1). Depending on nitrogen dose and irrigation regime, total phenolic content ranged from 30.99 to 40.68 mg GAE g^− 1^. The highest phenolic content was recorded under I_0_ conditions, whereas a regular decrease was observed with increasing irrigation regime. Among nitrogen treatments, the N_240_ dose resulted in the highest phenolic content. The surface graph showed that total phenolic content reached its maximum in combinations of low and moderate to high nitrogen doses (Fig. 3). In contrast, phenolic accumulation was significantly reduced under high irrigation conditions.

### Antioxidant capacities

**Fig. 4:**
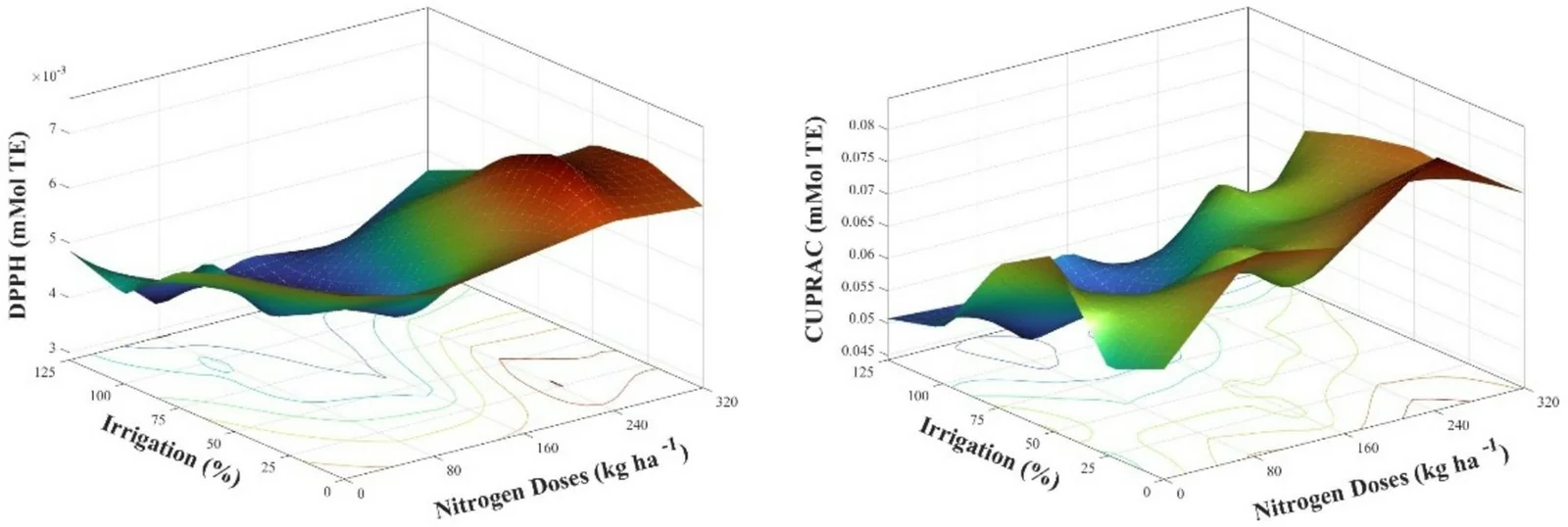
3D response surface showing the combined effects of nitrogen dose (kg ha⁻¹) and irrigation level (%) on antioxidant capacity (DPPH and CUPRAC) of sorghum silage

DPPH and CUPRAC antioxidant capacities were significantly affected by nitrogen dose and irrigation regime (*p* < 0.01; Table 1). For both parameters, the highest antioxidant capacities were determined under low irrigation conditions (I_0_-I_25_), followed by a gradual decrease with increasing irrigation regime. Regarding nitrogen doses, N_240_ and N_320_ treatments resulted in higher antioxidant capacities compared with the lower nitrogen doses. Surface graph showed that antioxidant capacity reached its maximum in combinations of deficit irrigation and moderate to high nitrogen doses (Fig. 4).

### Antioxidant enzyme activities

SOD, CAT, and POD activities were significantly affected by nitrogen dose, irrigation level, and their interaction (*p* < 0.01; Table 1). In general, antioxidant enzyme activities increased under water deficitconditions. Under full water restriction (I_0_), POD (5.06 U mg protein^-1^) and CAT (14.07 U mg protein^-1^) activities reached their highest levels and gradually decreased as irrigation regime increased. In contrast, SOD activity exhibited a different response pattern depending on irrigation regime and reached its maximum value under moderate water deficit conditions (I_75_). Relatively lower SOD activity was observed under both severe and lower water deficit conditions.

**Fig. 5:**
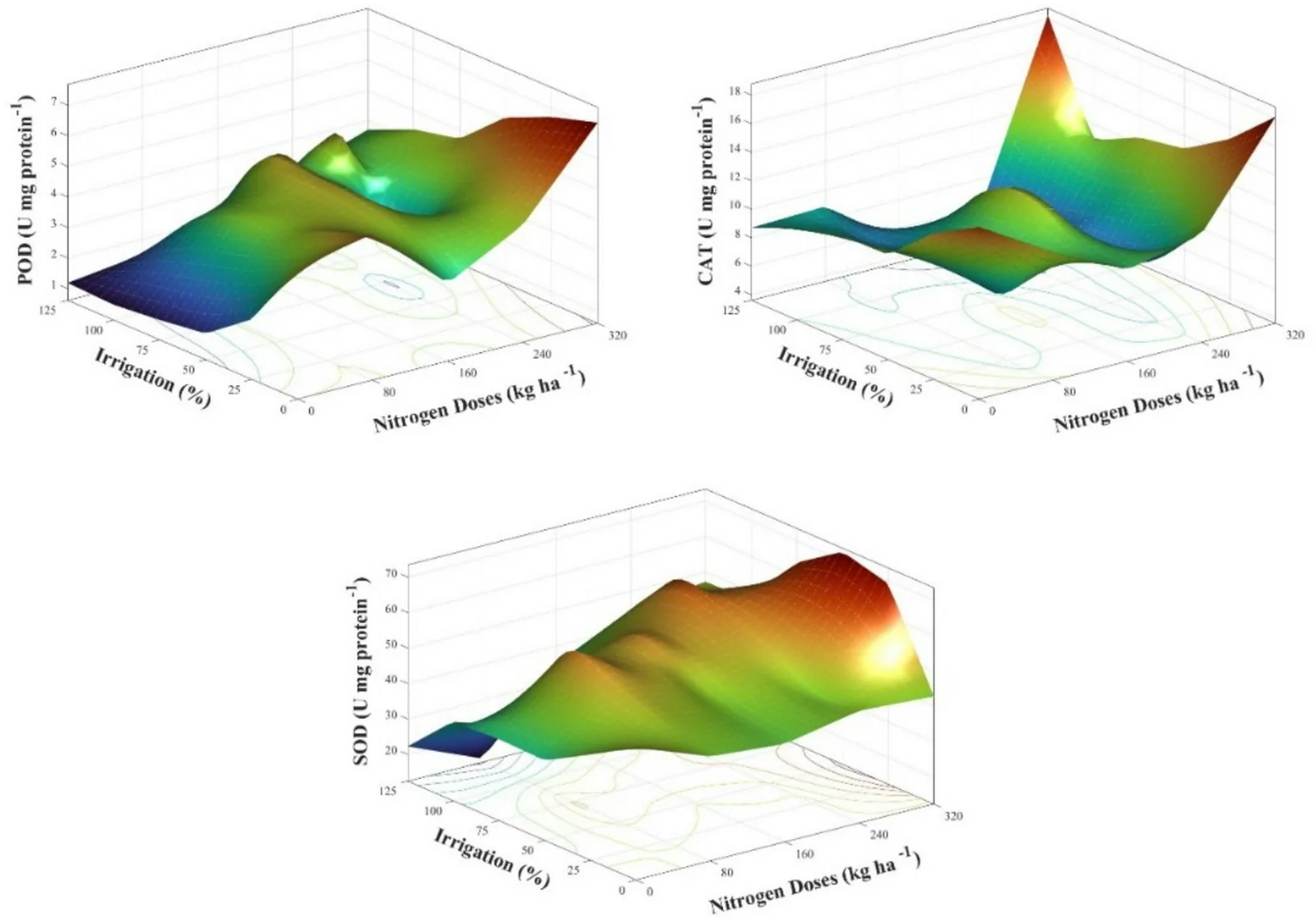
3D response surface showing the combined effects of nitrogen dose (kg ha⁻¹) and irrigation level (%) on antioxidant enzymes (POD, CAT and SOD) of sorghum silage. POD: peroxidase activity; CAT: catalase activity; SOD: superoxide dismutase activity. Enzyme activities are expressed as U mg protein⁻¹ fresh weight

Increasing nitrogen doses promoted antioxidant enzyme activities, particularly in SOD and POD activities. In general, medium and high nitrogen treatments resulted in higher enzyme activities, whereas low nitrogen doses were associated with comparatively reduced enzyme activity. Surface graph indicated that antioxidant enzyme activities were maximized in combinations of low to moderate irrigation regime and N_320_ nitrogen levels (Fig. 5).

### Oxidative stress indicators

Malondialdehyde (MDA) and hydrogen peroxide (H₂O₂) contents were significantly affected by nitrogen dose and irrigation regime (*p* < 0.01; Table 1). Oxidative stress indicators increased under water restriction conditions and gradually decreased with increasing irrigation regime. The highest MDA (0.481 nmol g^-1^) and H₂O₂ (70.06 µmol g^-1^) contents were determined under full water irrigation (I_0_), whereas lower levels were observed in the I_100_ and I_125_ treatments.

**Fig. 6:**
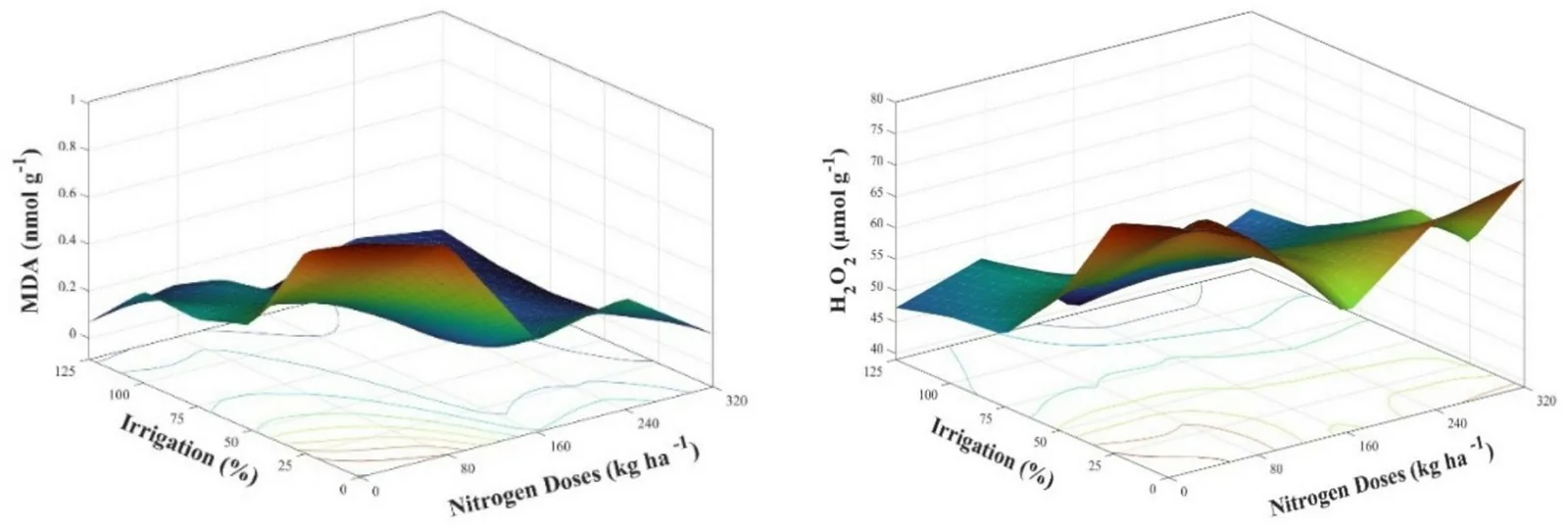
3D response surface showing the combined effects of nitrogen dose (kg ha⁻¹) and irrigation level (%) on oxidative stress (MDA and H_2_O_2_) of sorghum silage. MDA: malondialdehyde content (nmol g⁻¹ FW); H₂O₂: hydrogen peroxide content (µmol g⁻¹ FW)

Nitrogen applications had a regulatory effect on oxidative stress indicators. Increasing nitrogen doses reduced the accumulation of MDA and H₂O₂, particularly under low and moderate irrigation conditions, whereas oxidative stress indicators remained comparatively higher under low nitrogen treatments. Surface graph demonstrated that oxidative stress intensity increased with increasing water deficit condition, but this increase was partially alleviated by higher nitrogen dose (Fig. 6).

### Multivariate analysis

**Fig. 7:**
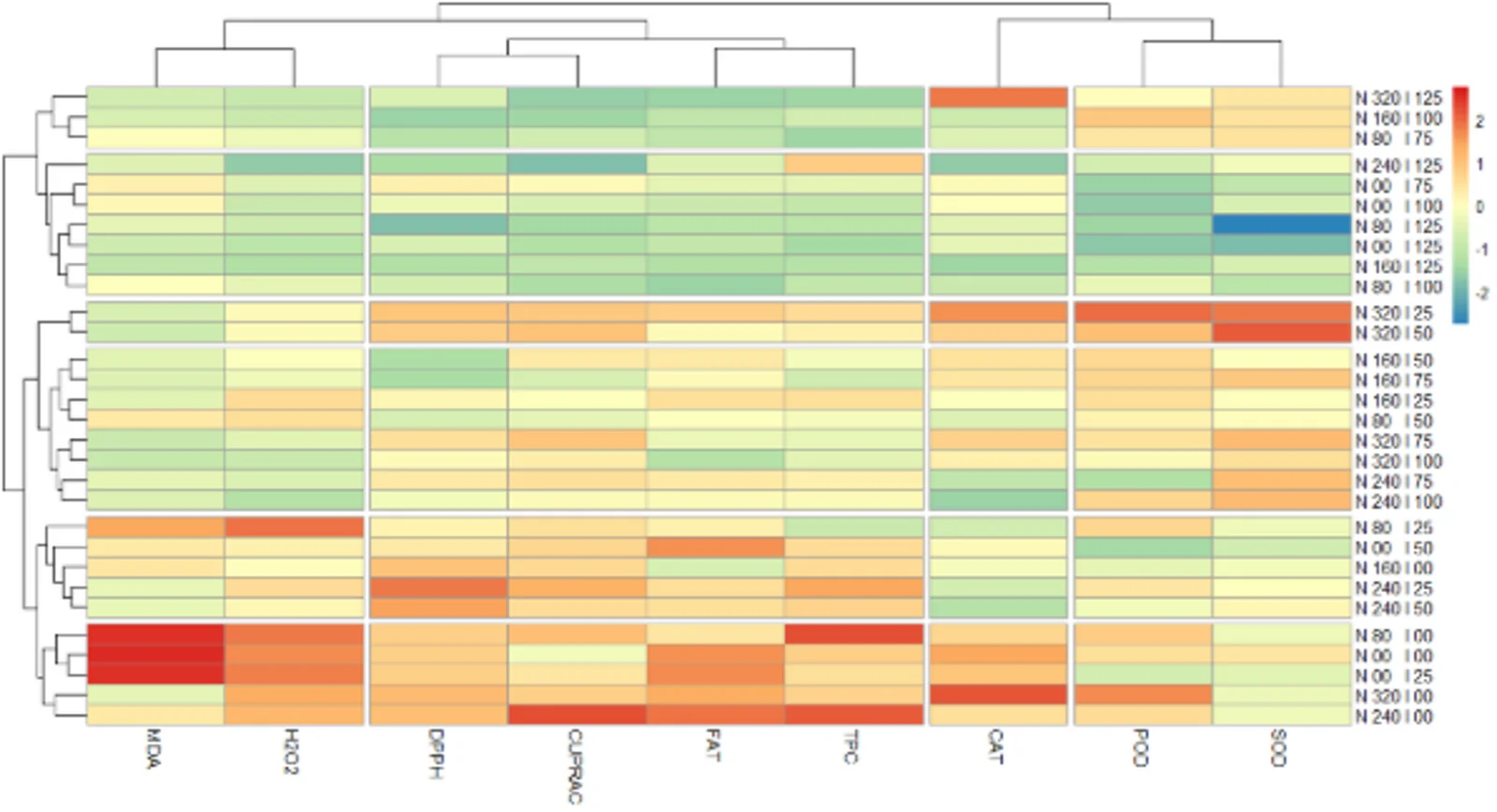
Hierarchical clustering heatmap showing the standardized biochemical responses of sorghum silage under different nitrogen and irrigation treatments. Red and blue indicate relatively higher and lower standardized values, respectively. FAT: Fat content, TPC: Total phenolic content, CUPRAC: Copper reduction capacity, DPPH: Free radical scavenging activity, POD: Peroxidase activity, CAT: Catalase activity, SOD: Superoxide dismutase activity, MDA: Malondialdehyde content, H₂O₂: Hydrogen peroxide content

Hierarchical clustering heat map analysis showed distinct clustering patterns among the biochemical parameters according to nitrogen dose and irrigation regime (Fig. 7). Following standardization, the variables were grouped into two main clusters. The first main cluster was divided into two sub-clusters. The first sub-cluster consisted of MDA and H₂O₂, which clustered closely together and exhibited similar color intensities. These oxidative stress indicators showed higher normalized values particularly under low irrigation and low nitrogen conditions, whereas comparatively lower values were observed under high irrigation combined with moderate to high nitrogen dose. The second sub-cluster included antioxidant capacity and metabolic quality parameters. Total phenolic content (TPC), DPPH, CUPRAC, and fat content (FAT) exhibited close similarity within the same sub-cluster. These parameters were found to show a tendency to increase together, particularly in specific nitrogen-irrigation combinations, and their color intensities showed parallelism. Antioxidant enzyme activities (CAT, POD, and SOD) formed a separate second main cluster, partially diverging from phenolic content and chemical antioxidant capacity parameters. SOD and POD were located closer together on the dendrogram, whereas CAT showed a more central association. Application based hierarchical clustering demonstrated that low irrigation treatments (especially I_0_ and I_25_) clustered together, moderate irrigation treatments (I_50_-I_75_) formed an intermediate group, and high irrigation treatments (I_100_-I_125_) were clustered under a separate branch. Regarding nitrogen doses, N_0_ treatments clustered more closely with oxidative stress indicators, whereas N_240_ and N_320_ treatments were more closely related to antioxidant enzyme activities.

**Fig. 8:**
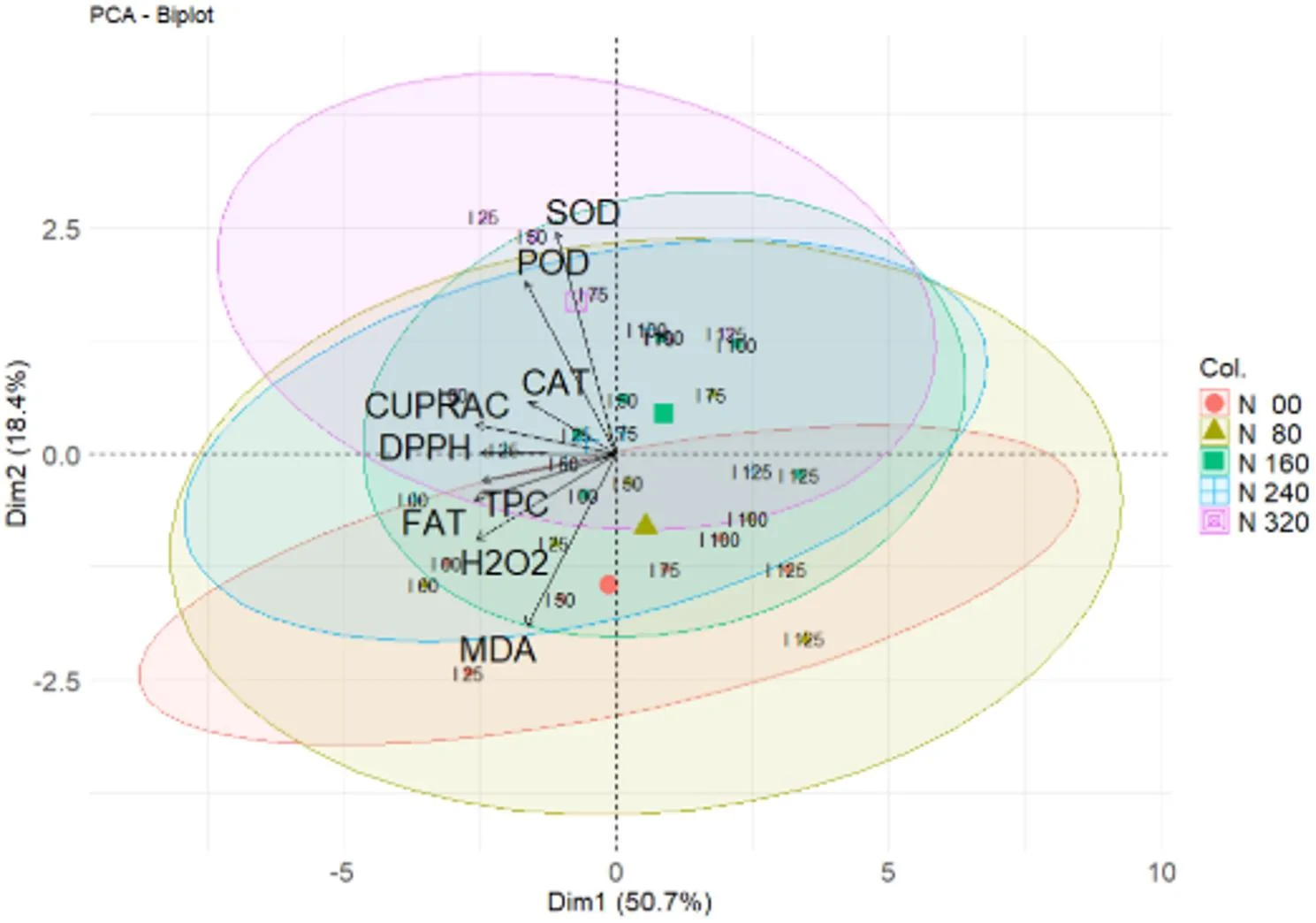
Principal component analysis (PCA) biplot illustrating multivariate relationships among biochemical parameters under different nitrogen and irrigation regimes in sorghum silage. Principal component 1 (PC1) and principal component 2 (PC2) explained 50.7% and 18.4% of the total variance, respectively. Variables positioned closely indicate positive associations, whereas variables located in opposite directions indicate negative relationships. FAT: Fat content, TPC: Total phenolic content, CUPRAC: Copper reduction capacity, DPPH: Free radical scavenging activity, POD: Peroxidase activity, CAT: Catalase activity, SOD: Superoxide dismutase activity, MDA: Malondialdehyde content, H₂O₂: Hydrogen peroxide content

Principal component analysis (PCA) revealed the multivariate relationships among the evaluated biochemical parameters along two principal axes (Fig. 8). The first principal component (PC1; Dim1) explained 50.7% of the total variance, whereas the second principal component (PC2; Dim2) explained 18.4%. Together, the two components collectively explained 69.1% of the total variance. Variables associated with antioxidant capacity and metabolic quality (TPC, DPPH, CUPRAC, and FAT) were primarily distributed along the PC1 axis, indicating that moderate water restriction supported antioxidant-related metabolic activity. In contrast, PC2 mainly reflected the enzymatic antioxidant defense response, largely driven by SOD and POD activities, particularly under increased nitrogen doses. A distinct separation was observed along the PC1 axis in the biplot distribution. TPC, DPPH, CUPRAC, and FAT were located in the negative direction of PC1, whereas high irrigation treatments (especially I_100_ and I_125_) were concentrated in the positive PC1 direction. Oxidative stress indicators (MDA and H₂O₂) were located in the negative PC1 and negative PC2 regions, separating them from the antioxidant related parameters. Along the PC2 axis, antioxidant enzyme activities showed distinct positioning patterns. In particular, SOD and POD showed high loading values in the positive PC2 direction, whereas CAT exhibited a more central distribution. Samples from the N_320_ treatment showed a wider distribution in the positive PC2 direction. Overall, PCA results indicated that TPC and antioxidant capacity parameters clustered along the same axis; antioxidant enzyme activities were separated along the second axis, and oxidative stress indicators occupied a distinct region of the PCA plane. The clear separation of oxidative stress indicators from antioxidant capacity parameters further suggests that stress intensity and antioxidant defense responses were regulated through partially distinct physiological mechanisms. Moreover, the clustering of antioxidant enzyme activities under moderate to high nitrogen treatments indicates that nitrogen doses contribute to strengthening the enzymatic ROS scavenging system under water deficitconditions.

**Fig. 9:**
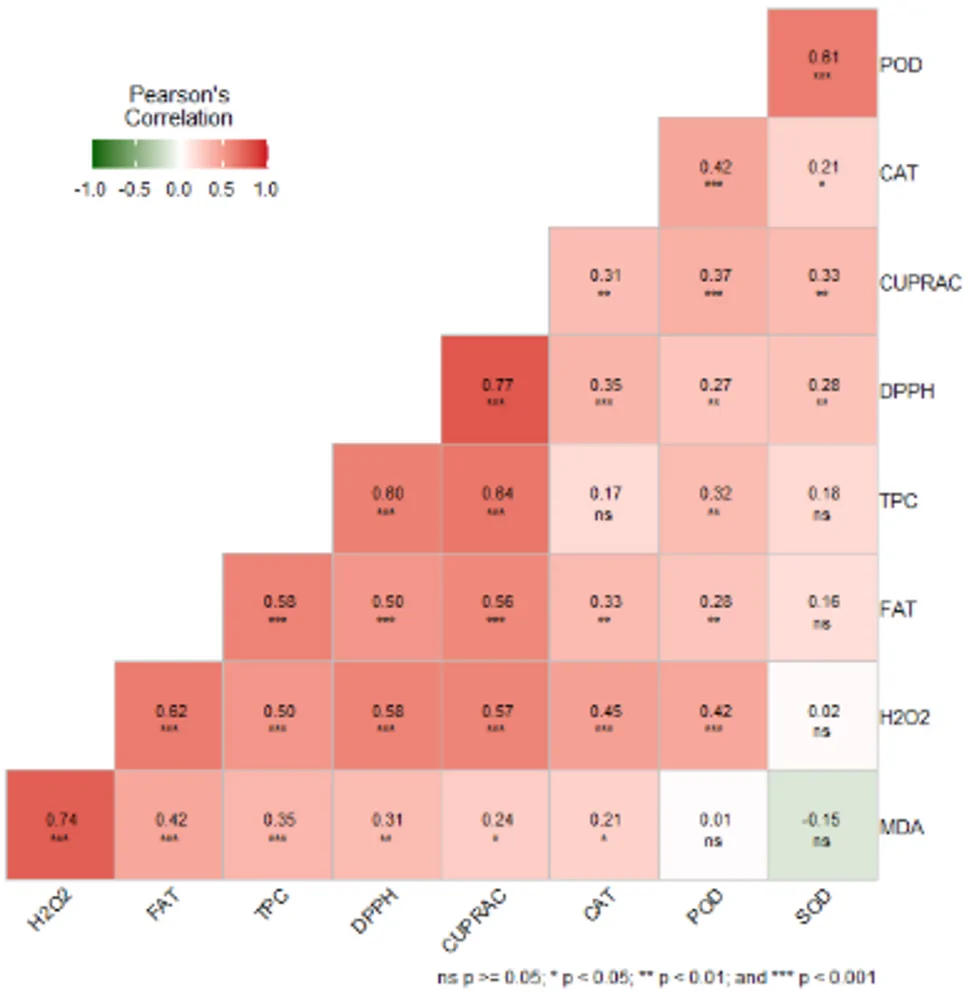
Pearson correlation matrix showing relationships among biochemical parameters measured in sorghum silage under different nitrogen and irrigation treatments. Positive correlations are indicated in green and negative correlations in red, with color intensity corresponding to correlation strength. FAT: Fat content, TPC: Total phenolic content, CUPRAC: Copper reduction capacity, DPPH: Free radical scavenging activity, POD: Peroxidase activity, CAT: Catalase activity, SOD: Superoxide dismutase activity, MDA: Malondialdehyde content, H₂O₂: Hydrogen peroxide content

Pearson correlation analysis showed significant relationships among the biochemical parameters examined (Fig. 9). A strong positive correlation was observed between oxidative stress indicators (MDA and H_2_O_2_) (*r* = 0.74). TPC showed a strong positive correlation with DPPH (*r* = 0.64) and moderate positive correlation with CUPRAC (*r* = 0.32). The correlation coefficient between DPPH and CUPRAC was calculated as *r* = 0.35. Among antioxidant enzyme activities, POD and CAT showed a positive correlation (*r* = 0.61). In addition, CUPRAC showed positive correlations with CAT (*r* = 0.37) and POD (*r* = 0.33). Although comparatively lower, the correlations of DPPH with CAT (*r* = 0.27) and POD (*r* = 0.28) were also statistically significant. The positive correlations observed between oxidative stress indicators and antioxidant defense parameters support the coordinated activation of protective mechanisms under stress conditions. Specifically, the strong correlation between MDA and H₂O₂ indicates that lipid peroxidation and ROS accumulation progressed simultaneously under water deficit conditions, whereas positive correlations among antioxidant enzymes and antioxidant capacity parameters suggest the activation of an integrated defense response against oxidative damage.

## Discussion

Lipid content and fatty acid composition in plant tissues are highly responsive to environmental factors that regulate carbon allocation and metabolic priorities. Previous studies in forage crops have demonstrated that harvest stage, developmental status, and climatic factors significantly alter lipid accumulation and fatty acid composition [38, 39]. In addition, membrane lipid restructuring during plant maturation contributes to species specific differences in fatty acid [40, 41].

The predominance of triacylglycerols and linoleic acid in sorghum highlights the potential importance of this species as a source of essential fatty acids. In the present study, the preservation of oleic, linoleic, and γ-linolenic acids after fermentation suggests that lipid oxidation remained limited under appropriate fermentation conditions. Although lipolysis and fatty acid oxidation may occur during wilting prior to fermentation, efficient compaction and anaerobic fermentation are known to preserve lipid stability [42]. These findings indicate that sorghum silage may serve not only as an important energy source for ruminants but also as a valuable functional lipid composition.

Nitrogen and irrigation management regulate lipid biosynthesis primarily through their effects on carbon allocation. Moderate nitrogen levels may promote carbon allocation toward lipid synthesis by enhancing photosynthetic capacity, whereas excessive nitrogen levels can redirect carbon metabolism toward protein synthesis at the shifting lipid accumulation. In sorghum, it has been reported that increasing nitrogen doses alter grain oil content and fatty acid composition, while excessive nitrogen application may suppress lipid accumulation [43]. Because studies directly evaluating oil content and fatty acid composition in sorghum silage are limited, the present findings were interpreted in relation to stress physiology and nutritional studies conducted in maize and other forage crops. Similar responses have been reported in maize, where nitrogen and irrigation regimes significantly influence crude oil content and fatty acid composition, while linoleic and oleic acids remain the predominant fatty acids [44, 45]. Water deficit conditions may suppress acetyl-CoA production and reducing power production by limiting stomatal conductance and CO₂ assimilation, thereby altering carbon metabolism in C4 plants [18, 19]. The changes observed in lipid content and fatty acid composition in present study are consistent with this physiological framework and demonstrate that water-nitrogen interactions dynamically regulate lipid metabolism. The increase in specific unsaturated fatty acids under deficit irrigation conditions may also represent an adaptive response aimed at maintaining membrane and cellular stability under water deficit. Previous studies have shown that drought stress can stimulate membrane lipid remodeling and increase the proportion of unsaturated fatty acids to preserve membrane functionality under oxidative stress conditions [46, 47]. Moderate water limitation may therefore promote selective accumulation of oleic and linoleic acids as part of stress adaptation mechanisms associated with membrane stabilization and ROS tolerance.

From an agronomic perspective, these findings are particularly relevant for forage production systems in semi-arid and water-limited regions, where irrigation water availability is increasingly constrained. The present results suggest that moderate water deficit combined with appropriate nitrogen management may improve silage quality by preserving functional fatty acids and enhancing antioxidant-related metabolites without severely compromising physiological stability. The preservation of essential unsaturated fatty acids, particularly linoleic and *α*-linolenic acids, is critically important for ruminant nutrition because these compounds serve as precursors for conjugated linoleic acid (CLA) synthesis in milk fat [17]. Previous studies have been shown that forage and silage based diates increase CLA levels in milk fat [48–50]. Therefore, the preservation of unsaturated fatty acids in sorghum silage represents an important advantage in terms of forage quality and nutritional value. Furthermore, optimizing nitrogen-water management strategies may contribute not only to sustainable forage production but also to improving the nutritional and functional quality of silage under climate stress conditions.

In the present study, the increase in total phenolic content under water deficit conditions indicates that phenylpropanoid metabolism was actively under stress conditions. Phenolic compounds play a cenral role in plant defense against oxidative stress through their direct reactive oxygen species (ROS) scavenging activity [14]. Sorghum is naturally rich in phenolic compounds, and stress conditions are known to further enhance their accumulation [13]. Similar responses have also been reported in maize, where drought and nitrogen limitation stimulate the accumulation of flavonoids and phenolic acids as part of an adaptive response that mitigates oxidative damage caused by ROS [6].

Nitrogen availability may further influence oxidative stress regulation through its role in amino acid metabolism, protein synthesis, and enzymatic antioxidant production. Adequate nitrogen supply supports chlorophyll synthesis, photosynthetic electron transport, and the generation of metabolic energy required for antioxidant defense activation. Moreover, nitrogen-dependent metabolic pathways are closely associated with phenylalanine availability, which directly regulates phenylpropanoid biosynthesis and phenolic compound accumulation. The effect of nitrogen levels on phenolic accumulation can be explained by the carbon-nitrogen balance theory. High nitrogen level may favor growth related metabolism while suppressing secondary metabolite synthesis, whereas moderate nitrogen availability may support carbon allocation toward defense related metabolism [51, 52]. The increased phenolic accumulation observed at moderate to high nitrogen doses in the present study is consistent with this theoretical framework. Consequently, nitrogen management may simultaneously modulate phenolic metabolism, antioxidant enzyme activity, and ROS detoxification capacity under drought stress conditions. Phenolic compounds are important not only for stress tolerance but also for improving the functional quality of feed and food products. Sorghum phenolic have been associated with potential health promoting properties due to their strong antioxidant capacity [53–56]. The present study suggests that optimizing nitrogen and irrigation management to enhance phenolic content may also improve the functional feed quality of sorghum silage.

The parallel variation observed between total phenoliccontent and DPPH/CUPRAC antioxidant capacities indicates that antioxidant capacity in sorghum silage was largely associated with phenolic compounds. The phenolic in sorghum possess high free radical scavenging activity and contribute substantially to the total antioxidant capacity [57]. Activation of the phenylpropanoid pathway under abiotic stress conditions, such as drought stress, supports the direct neutralization of ROS by increasing flavonoid and phenolic acid synthesis [14]. Increased phenolic accumulation accompanied by enhanced antioxidant capacity under drought conditions has also been reported in sweet sorghum [58]. Consistent with this literature, the present study demonstrates that phenolic-based antioxidant responses were enhanced under deficit irrigation conditions.

Under abiotic stress conditions, reduced cellular water potential and ion imbalance stimulate excessive ROS production in chloroplasts and mitochondria, resulting in membrane damage and macromolecular degradation [15, 59, 60]. The accumulation of superoxide radicals (O_2_^−^) and H_2_O_2_ promotes membrane lipid peroxidation and compromises cellular integrity. In this process, SOD, CAT, and POD constitute a coordinated antioxidant defense system. SOD catalyzes the conversion of superoxide radicals into H_2_O_2_, whereas CAT and POD subsequently detoxify H₂O₂ into water and oxygen, thereby reducing oxidative pressure [61, 62]. In the present study, increased SOD, CAT, and POD activities, especially under moderate stress conditions, indicates that the rapid activation of enzymatic defense system against ROS accumulation. Under moderate water restriction, ROS production may have functioned as a signaling mechanism that stimulated antioxidant defense activation and increased SOD synthesis. However, under severe stress conditions, excessive ROS accumulation may exceed the detoxification capacity of the antioxidant system and partially suppress enzymatic activity through oxidative damage to proteins and membrane structures. A similar biphasic response of SOD activity under drought stress has been reported in many plant species, where moderate stress increased antioxidant defense capacity, whereas severe stress resulted in enzymatic inhibition and metabolic disruption [63, 64]. It has been reported in a study on maize that drought stress significantly increases SOD, CAT, APX, and GR activities, thereby protecting cellular integrity through the suppression lipid peroxidation [2]. Similarly, drought and salinity stress have been reported to antioxidant enzyme activities in various plant species, contributing to improved stress tolerance [58, 65]. Water and nitrogen deficiency in maize are known to disrupt metabolic stability and intensify stress responses, whereas optimal nutritional conditions support cellular homeostasis and physiological resilience [44]. These studies indicate that the enzymatic antioxidant defense system plays a central role in stress adaptation in C4 plants.

MDA and H_2_O_2_ levels are widely accepted as biochemical indicators of oxidative damage and lipid peroxidation [16, 66]. Increased accumulation of these parameters under high stress conditions indicates that membrane lipids are subjected to oxidative deterioration. In the present study, decreased MDA levels accompanied by enhanced antioxidant enzyme activities at appropriate nitrogen doses suggests that nitrogen management may improve oxidative stress tolerance. The positive effects of nitrogen on photosynthetic capacity and metabolic energy production may contribute to maintaining the sustainability of antioxidant defense system [2]. Overall, it indicate that antioxidant capacity and enzymatic defense systems in sorghum silage function coordinately to limit ROS-induced damage and that this defense network can be modulated through nitrogen and irrigation management.

Although increased MDA and H_2_O_2_ accumulation under severe water restriction reflected enhanced oxidative stress, elevated SOD, POD, and CAT activities demonstrated the activation of enzymatic antioxidant defense mechanisms in response to excessive ROS formation. However, under severe stress conditions, antioxidant defense activation was not always sufficient to completely prevent oxidative damage, resulting in the simultaneous increase of oxidative stress markers and antioxidant enzyme activities. This coordinated response suggests that sorghum plants attempt to mmaintain redox homeostatis through a dynamic balance between ROS generation and ROS scavenging. Similar stress-induced interactions between ROS accumulation and antioxidant defense activation under abiotic stress conditions have also been widely reported in sorghum and other cereal crops [64, 67].

Hierarchical clustering heat map analysis revealed biologically related clustering patterns among the evaluated biochemical parameters. The clustering of total phenolic content together with DPPH and CUPRAC indicates that the non-enzymatic antioxidant system functions in an integrated manner. In contrast, the distinct positioning of MDA and H_2_O_2_ on a seperate axis suggests that oxidative stress indicators were differentiated from antioxidant defense related parameters. The close clustering of antioxidant enzymes (SOD, CAT, and POD) with total phenolic content further suggests coordinated interactions between enzymatic and non-enzymatic antioxidant defense systems.

Multivariate analyses indicate that stress tolerance is regulated not by a single biochemical parameter but through integrated redox networks [68, 69]. The large proportion of variance explained by the conrast between antioxidant defense capacity and oxidative stress indicators in PCA analysis demonstrates that nitrogen-water regimes substantially reshaped oxidative and metabolic responses. Similar PCA-based studies in sorghum and maize have shown that stress tolerance is largely organized along antioxidant defense related metabolic axis [2, 70, 71]. Previous studies conducted by Kaplan et al. [44, 45] in maize and sorghum demonstrated that nitrogen and irrigation management comprehensively restructure metabolic profiles and generate distinct physiological response patterns, which closely parallel the PCA distribution observed in the present study.

The strong positive correlations observed between total phenolic content and DPPH/CUPRACantioxidant capacities confirm that phenolic compounds are major contributors to antioxidant capacity. In contrast, the negative correlations between MDA and antioxidant enzyme activities indicate the effectiveness of antioxidant defense system in limiting lipid peroxidation. This dynamic intraction between ROS production and antioxidant defense systems has been widely recognized as a key determinant of stress tolerance under abiotic stress conditions [2, 14, 72].

## Conclusion

This study demonstrated that nitrogen dose and irrigation regime not only affect growth and yield parameters in sorghum silage but also substantially regulate lipid metabolism and antioxidant defense systems. The results showed that nitrogen-water interactions significantly influenced crude oil content, fatty acid composition, antioxidant capacity, and enzymatic defense responses. Water deficit conditions promoted increase in crude oil content, total phenolic content, and antioxidant capacity, whereas excessive irrigation reduced these biochemical parameters. In contrast, moderate to high nitrogen treatments partially alleviated oxidative stress by enhancing antioxidant enzyme activities and limiting MDA and H_2_O_2_ accumulation. In particular, the combination of moderate water restriction (I_50_-I_75_) with medium to high nitrogen doses (N_240_-N_320_) provided the most balanced biochemical response by improving antioxidant defense capacity while limiting oxidative damage. Multivariate analyses demonstrated that stress adaptation in sorghum silage is regulated through coordinated interactions among lipid metabolism, antioxidant defense systems, and oxidative stress indicators rather than through individual biochemical parameters alone. The clear distinct between oxidative stress parameters and antioxidant related variables in PCA and clustering analyses highlights the complex physiological regulation underlying nitrogen-water interactions under stress conditions. The results suggest that optimized nitrogen fertilization combined with controlled deficit irrigation may improve both silage quality and oxidative stability under semi-arid production conditions. These strategies may contribute to more sustainable forage production by improving functional feed quality while reducing excessive water use. Future studies should further investigate the long-term effects of nitrogen–water management on silage fermentation quality, digestibility, and animal performance under different environmental conditions.

## Supplementary Information

Supplementary Material 1.

## Data Availability

Data will be made available on request. Contacts: [mehmetalagoz@isparta.edu.tr](mailto: mehmetalagoz@isparta.edu.tr) (Mehmet ALAGÖZ).
