# Supplementary material for: Nitrogen-water management regimes modulate fatty acid profile, antioxidant defense system, and oxidative status in sorghum (Sorghum bicolor L.) silage
Source: BMC Plant Biol. 2026 Jul 31;26:1439. doi: 10.1186/s12870-026-09594-4 (PMC13495516; doi:10.1186/s12870-026-09594-4)
Supplement: Supplementary file 1 [file 12870_2026_9594_MOESM1_ESM.docx]

Supplental Table 1. Results of analysis of variance of the traits determined.

| **Sources  of  Variations** | **df** | **Fat Content  (%)** | **Total Phenolic Content  (mg GAE g^-1^ DM)** | **DPPH  (mMol TE)** | **CUPRAC (mMol TE)** | **POD  (U mg protein^-1^)** | **CAT  (U mg protein^-1^)** | **SOD  (U mg protein^-1^)** | **MDA  (nmol g^-1^)** | **H_2_O_2_  (µmol g^-1^)** |
| --- | --- | --- | --- | --- | --- | --- | --- | --- | --- | --- |
| **Block** | 2 | ns | ns | ns | ns | ns | ns | ns | ns | ns |
| **Nitrogen (N)** | 4 | * | ** | ** | ** | ** | ** | ** | ** | ** |
| **Error 1** | 8 |  |  |  |  |  |  |  |  |  |
| **Irrigation Level (IL)** | 5 | ** | ** | ** | ** | ** | ** | ** | ** | ** |
| **N x IL intr.** | 20 | ns | ** | ** | ** | ** | ** | ** | ** | ** |
| **Error 2** | 50 |  |  |  |  |  |  |  |  |  |
| **Corrected Total** | 89 |  |  |  |  |  |  |  |  |  |
| **Coeff Var** |  | 17.47 | 7.26 | 4.17 | 5.99 | 9.23 | 5.99 | 8.65 | 16.81 | 3.94 |

Supplental Table 2. Average values for the interaction between nitrogen doses and irrigation levels. (Presented as a 3D surface graph in the article).

| **Irrigation Level** | **Fat Content (%)** | | | | |  | **Irrigation Level** | **Total Phenolic Content (mg GAE g^-1^ DM)** | | | | | |
| --- | --- | --- | --- | --- | --- | --- | --- | --- | --- | --- | --- | --- | --- |
|  | **Nitrogen Doses** | | | | |  |  | **Nitrogen Doses** | | | | | |
|  | **N_0_** | **N_80_** | **N_160_** | **N_240_** | **N_320_** |  |  | **N_0_** | **N_80_** | **N_160_** | **N_240_** | **N_320_** |  |
| **I_0_** | 0,333 | 0.264 | 0.203 | 0.347 | 0.315 |  | **I_0_** | 38.49 bc | 44.95 a | 37.36 bd | 44.34 a | 38.25 bc |  |
| **I_25_** | 0.337 | 0.253 | 0.270 | 0.268 | 0.284 |  | **I_25_** | 37.29 bd | 30.90 fı | 37.33 bd | 41.13 ab | 37.58 bd |  |
| **I_50_** | 0.332 | 0.237 | 0.260 | 0.268 | 0.243 |  | **I_50_** | 37.52 bd | 34.35 cg | 34.15 cg | 38.25 bc | 36.04 ce |  |
| **I_75_** | 0.214 | 0.181 | 0.244 | 0.258 | 0.220 |  | **I_75_** | 33.01 dı | 28.16 ı | 31.42 eı | 35.97 ce | 33.25 dh |  |
| **I_100_** | 0.190 | 0.150 | 0.180 | 0.243 | 0.170 |  | **I_100_** | 30.59 fı | 30.51 fı | 31.85 eı | 35.16 cf | 32.82 dı |  |
| **I_125_** | 0.184 | 0.177 | 0.169 | 0.209 | 0.153 |  | **I_125_** | 28.42 hı | 30.02 gı | 29.52 gı | 38.66 bc | 28.36 hı |  |
|  | | | | | | | | | | | | | |
| **Irrigation Level** | **DPPH (mMol TE)** | | | | |  | **Irrigation Level** | **CUPRAC (mMol TE)** | | | | | |
|  | **Nitrogen Doses** | | | | |  |  | **Nitrogen Doses** | | | | | |
|  | **N_0_** | **N_80_** | **N_160_** | **N_240_** | **N_320_** |  |  | **N_0_** | **N_80_** | **N_160_** | **N_240_** | **N_320_** |  |
| **I_0_** | 0.0061 cd | 0.0061 cd | 0.0063 c | 0.0064 bc | 0.0064 bc |  | **I_0_** | 0.060 gj | 0.074 bc | 0.069 bf | 0.085 a | 0.071 be |  |
| **I_25_** | 0.0061 cd | 0.0055 ef | 0.0054 eg | 0.0071 a | 0.0063 c |  | **I_25_** | 0.066 dg | 0.068 cf | 0.062 fı | 0.075 b | 0.072 be |  |
| **I_50_** | 0.0057 ef | 0.0046 jk | 0.0040 l | 0.0067 b | 0.0061 cd |  | **I_50_** | 0.069 bf | 0.058 hk | 0.066 dg | 0.068 cf | 0.073 bd |  |
| **I_75_** | 0.0055 ef | 0.0042 l | 0.0040 l | 0.0057 ef | 0.0058 de |  | **I_75_** | 0.063 fh | 0.055 jm | 0.056 ıl | 0.068 cf | 0.072 be |  |
| **I_100_** | 0.0050 hj | 0.0045 k | 0.0038 lm | 0.0051 gı | 0.0053 fh |  | **I_100_** | 0.056 ıl | 0.049 mo | 0.048 no | 0.063 fh | 0.065 eg |  |
| **I_125_** | 0.0046 jk | 0.0035 m | 0.0041 l | 0.0040 l | 0.0047 ık |  | **I_125_** | 0.050 lo | 0.048 no | 0.052 kn | 0.044 o | 0.046 no |  |
|  | | | | | | | | | | | | | |
| **Irrigation Level** | **POD (U mg protein^-1^)** | | | | |  | **Irrigation Level** | **CAT (U mg protein^-1^)** | | | | | |
|  | **Nitrogen Doses** | | | | |  |  | **Nitrogen Doses** | | | | | |
|  | **N_0_** | **N_80_** | **N_160_** | **N_240_** | **N_320_** |  |  | **N_0_** | **N_80_** | **N_160_** | **N_240_** | **N_320_** |  |
| **I_0_** | 4.87 cf | 5.43 bd | 3.22 jk | 4.96 ce | 6.80 a |  | **I_0_** | 15.86 c | 13.11 df | 10.05 ıj | 12.53 eg | 18.80 a |  |
| **I_25_** | 2.67 k | 5.16 be | 4.81 df | 4.65 eg | 7.33 a |  | **I_25_** | 14.21 d | 7.70 ln | 10.55 hı | 8.07 km | 16.82 bc |  |
| **I_50_** | 1.47 lm | 4.28 fh | 5.04 ce | 3.60 ıj | 5.75 b |  | **I_50_** | 10.86 hı | 8.50 km | 12.38 eg | 6.07 op | 13.44 de |  |
| **I_75_** | 1.22 lm | 4.61 eh | 5.08 ce | 1.76 l | 4.70 eg |  | **I_75_** | 10.89 hı | 8.66 k | 12.08 fg | 6.73 no | 13.38 de |  |
| **I_100_** | 1.07 m | 3.26 jk | 5.53 bc | 5.15 be | 4.07 gı |  | **I_100_** | 10.42 ı | 7.36 mn | 7.46 mn | 4.80 q | 11.62 gh |  |
| **I_125_** | 0.99 m | 1.41 lm | 1.85 l | 2.74 k | 3.97 hı |  | **I_125_** | 9.10 jk | 8.85 kl | 5.14 pq | 4.41 q | 17.60 b |  |
|  | | | | | | | | | | | | | |
| **Irrigation Level** | **SOD (U mg protein^-1^)** | | | | |  | **Irrigation Level** | **MDA (nmol g^-1^)** | | | | | |
|  | **Nitrogen Doses** | | | | |  |  | **Nitrogen Doses** | | | | | |
|  | **N_0_** | **N_80_** | **N_160_** | **N_240_** | **N_320_** |  |  | **N_0_** | **N_80_** | **N_160_** | **N_240_** | **N_320_** |  |
| **I_0_** | 50.40 cf | 42.05 gj | 42.68 ej | 41.65 hj | 41.25 hj |  | **I_0_** | 0.806 a | 0.797 a | 0.332 c | 0.323 c | 0.148 ef |  |
| **I_25_** | 39.01 hk | 42.13 fj | 44.39 dı | 44.39 dı | 67.97 a |  | **I_25_** | 0.794 a | 0.552 b | 0.132 eg | 0.165 e | 0.100 eh |  |
| **I_50_** | 35.81 jk | 44.99 dı | 44.51 dı | 46.63 dh | 71.30 a |  | **I_50_** | 0.323 c | 0.326 c | 0.135 eg | 0.158 e | 0.068 gı |  |
| **I_75_** | 32.65 k | 50.62 ce | 56.40 bc | 58.15 bc | 59.23 b |  | **I_75_** | 0.294 cd | 0.242 d | 0.129 eg | 0.155 e | 0.048 hı |  |
| **I_100_** | 37.17 ık | 31.87 k | 50.76 ce | 59.00 b | 51.52 bd |  | **I_100_** | 0.274 cd | 0.239 d | 0.106 eh | 0.129 eg | 0.042 hı |  |
| **I_125_** | 22.28 l | 10.95 m | 37.00 ık | 42.65 ej | 50.22 cg |  | **I_125_** | 0.065 gı | 0.155 e | 0.016 ı | 0.126 eg | 0.074 fı |  |
|  | | | | | | | | | | | | | |
| **Irrigation Level** | **H_2_O_2_ (µmol g^-1^)** | | | | |  |  |  |  |  |  |  | |
|  | **Nitrogen Doses** | | | | |  |  |  |  |  |  |  | |
|  | **N_0_** | **N_80_** | **N_160_** | **N_240_** | **N_320_** |  |  |  |  |  |  |  |  |
| **I_0_** | 74.39 ab | 75.91 a | 58.64 ef | 69.85 c | 71.52 bc |  |  |  |  |  |  |  |  |
| **I_25_** | 75.45 a | 76.36 a | 63.48 d | 63.48 d | 58.79 ef |  |  |  |  |  |  |  |  |
| **I_50_** | 60.30 de | 63.18 d | 57.73 eg | 59.55 df | 59.09 ef |  |  |  |  |  |  |  |  |
| **I_75_** | 53.03 hj | 55.45 fh | 55.91 fh | 52.42 hj | 53.33 hj |  |  |  |  |  |  |  |  |
| **I_100_** | 49.70 jl | 54.24 gı | 49.55 jl | 46.67 kl | 50.00 jl |  |  |  |  |  |  |  |  |
| **I_125_** | 47.88 kl | 50.61 ık | 45.91 l | 42.12 m | 49.09 jl |  |  |  |  |  |  |  |  |
